# AI-driven dynamic orthodontic treatment management: personalized progress tracking and adjustments—a narrative review

**DOI:** 10.3389/fdmed.2025.1612441

**Published:** 2025-08-01

**Authors:** Xuanchi Guo, Yuhan Shao

**Affiliations:** ^1^ Department of Dental Medicine, Shandong University, Jinan, Shandong, China; ^2^ State Key Laboratory of Oral Diseases & National Center for Stomatology & National Clinical Research Center for Oral Diseases, West China Hospital of Stomatology, Sichuan University, Chengdu, China

**Keywords:** orthodontics, digital treatment simulation, artificial intelligence, convolutional neural networks, patient engagement

## Abstract

Artificial intelligence (AI) is reconfiguring the orthodontic treatment paradigm through dynamic data-driven strategies. In this paper, we systematically review the multidimensional applications of AI in personalized treatment tracking, real-time decision support, and risk prediction, and reveal its core mechanisms to enhance clinical efficacy and patient experience. This review will focus on the fusion of AI-driven multimodal data analysis (e.g., cone-beam CT, intraoral scanning, and 3D facial images) and deep learning algorithms (e.g., convolutional neural networks) to elucidate the technological breakthroughs in key aspects such as tooth movement trajectory prediction and early detection of root resorption. Clinical practice has shown that AI has formed a complete closed loop of clinical application by optimizing the process of treatment plan development, realizing dynamic adjustment mechanisms, and enhancing patient compliance based on mobile medical platforms. Current research still needs to address core issues such as data privacy protection framework, algorithm interpretability enhancement, and multi-center validation. With the integration of interdisciplinary technology and the deepening of the research and development of intelligent orthodontic systems, AI will promote orthodontic diagnosis and treatment in the direction of more accuracy and personalization and ultimately realize the dual innovation of clinical decision-making mode and patient management strategy.

## 1 Introduction

Orthodontic treatment is a prolonged and adaptive process that typically spans months or even years, depending on the complexity of the case and the patient's response to treatment (1). Achieving the desired outcomes requires not only continuous monitoring but also frequent, precise adjustments to the treatment plan based on the patient's progress. Traditionally, orthodontic treatment planning and progress tracking have been heavily reliant on clinician expertise, periodic patient assessments, and manual comparisons of imaging data, such as x-rays, cephalometric analyses, and dental scans. While these methods have been fundamental in orthodontics, they come with several inherent limitations that can affect treatment outcomes (2).

One of the primary limitations is the subjectivity involved in decision-making. Clinicians must interpret data from various sources, which can lead to inconsistencies in treatment planning, especially when assessing subtle tooth movements or predicting long-term treatment effects (3). Furthermore, tracking progress over time can be challenging, particularly when changes are gradual or not easily noticeable through conventional methods (4). This is further compounded by the difficulty in predicting and mitigating potential complications, such as root resorption, periodontal disease, or orthodontic relapse, all of which can significantly affect the success of treatment if not identified early (5).

With the increasing complexity of modern orthodontic cases and the growing number of patients in care, it has become clear that traditional methods of treatment management, while valuable, need to evolve. There is a pressing need for a more efficient, data-driven, and predictive approach to orthodontic management that can improve the precision of treatment plans, streamline the monitoring process, and enable earlier detection of potential risks. Artificial Intelligence (AI) has emerged as a powerful tool to address these challenges, offering promising solutions for enhancing diagnosis, optimizing treatment planning, and providing real-time decision support (6).

By leveraging AI-driven algorithms, clinicians can now analyze vast amounts of patient data—from imaging scans to health records—allowing for more accurate, real-time assessments of treatment progres (7). These algorithms can not only track tooth movement with greater precision but also identify risks, such as root resorption and periodontal deterioration, before they reach a clinically significant stage. Furthermore, AI acts as an intelligent clinical assistant, helping orthodontists make patient-specific adjustments to the treatment plan, which can reduce treatment time, optimize outcomes, and minimize human error in clinical decision-making. This ultimately enhances the overall efficiency of orthodontic workflows, increases treatment predictability, and improves patient satisfaction (8).

This review delves into the challenges inherent in traditional orthodontic treatment management, investigates the potential of AI-driven systems to transform clinical workflows, and explores the benefits of integrating AI into real-time treatment tracking, risk prediction, and clinical decision-making. By analyzing current applications and exploring future possibilities, this paper aims to highlight the evolving role of AI in making orthodontic care more efficient, personalized, and proactive, ultimately leading to better patient outcomes and enhanced clinical practice.

**SCHEME 1:**
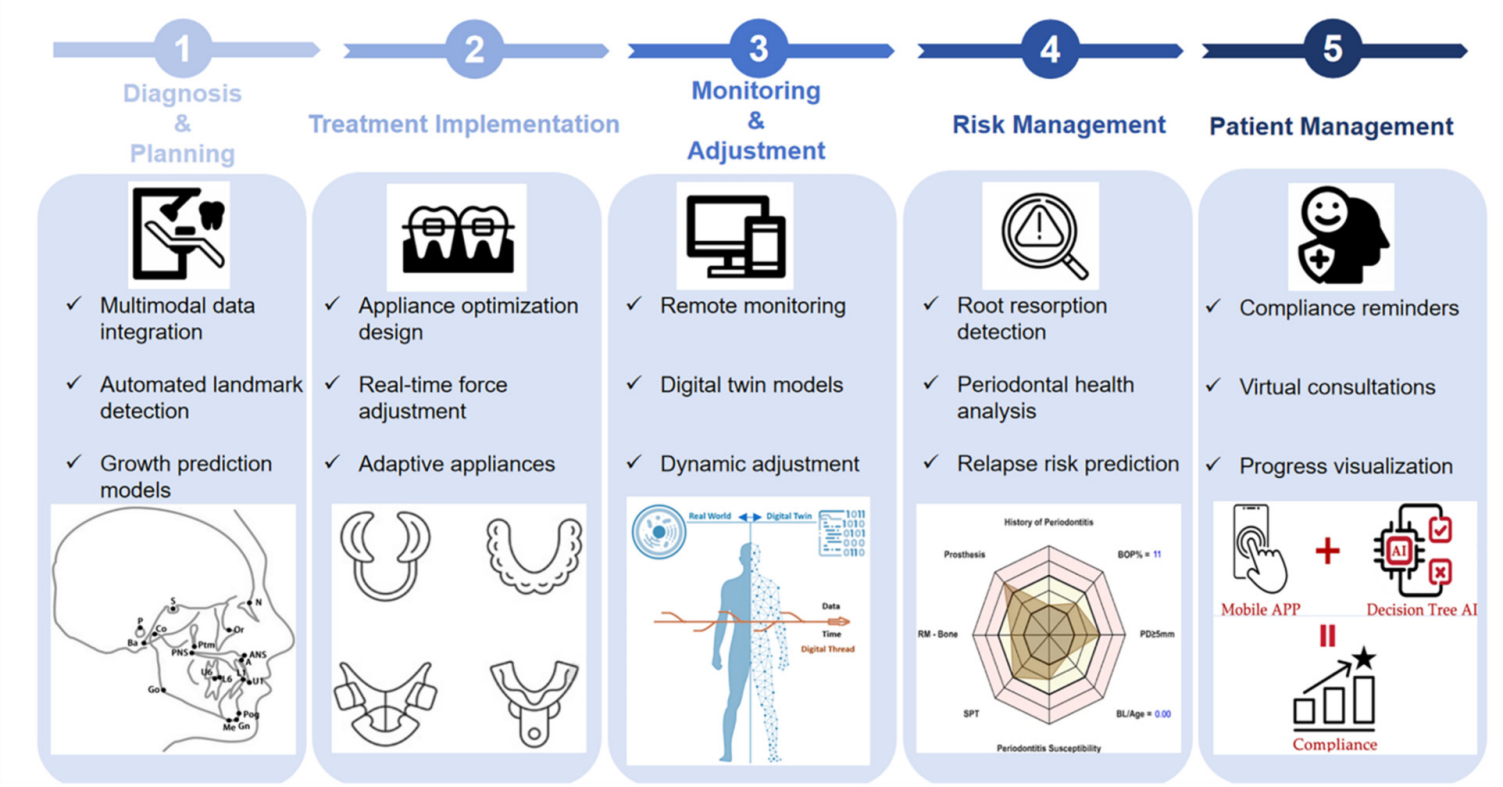
AI-augmented workflow in orthodontic treatment.

To guide our review of AI applications in orthodontic care, Scheme 1 presents a conceptual framework that organizes the clinical workflow into five AI-enhanced modules: diagnosis, treatment implementation, monitoring, risk management, and patient engagement. This framework provides the structural backbone for the following chapters.

## 2 Foundations of AI in orthodontics

AI has emerged as a transformative tool in orthodontics, enhancing diagnostic accuracy, predictive modeling, and real-time treatment monitoring. The foundation of AI in orthodontics lies in its ability to process and integrate multimodal data sources, including cephalometric x-rays, cone-beam computed tomography (CBCT), intraoral scans (IOS), 3D facial imaging, and patient medical records. By leveraging machine learning (ML) and deep learning (DL) algorithms, AI automates complex analyses, predicts treatment outcomes, and facilitates adaptive orthodontic interventions (9).

### 2.1 Multimodal data integration for orthodontic diagnostics

A core advantage of AI in orthodontics is its ability to synthesize diverse imaging and clinical data into a unified analytical framework. Traditional orthodontic assessments rely on separate 2D and 3D imaging modalities, requiring manual interpretation that is subject to interobserver variability. AI-powered systems overcome these limitations by integrating information from CBCT, IOS, and 3D facial surface imaging, enabling a more precise and comprehensive assessment of craniofacial structures and dental alignment. This capability is demonstrated in tools like Ceph-Net, which uses an attention-based stacked regression network to automatically detect cephalometric landmarks on scanned lateral cephalograms from children and adolescents with clinically acceptable accuracy (92.3% of landmarks within 2 mm error) (10).

Recent advancements in deep learning-based multimodal fusion systems have enabled AI to combine CBCT and IOS data for orthodontic treatment monitoring, allowing orthodontists to track root movement and jawbone remodeling without requiring repeated CBCT scans. Such systems have demonstrated high segmentation accuracy, achieving Dice coefficients of 94.1% for teeth and 94.4% for jawbones, facilitating treatment tracking (11).

Additionally, AI-driven cephalometric landmark detection using convolutional neural networks (CNNs) has significantly improved diagnostic consistency, reducing manual tracing errors and increasing efficiency in orthodontic analyses (12). AI has also been successfully applied to soft tissue segmentation, allowing for detailed assessments of gingival thickness, periodontal health, and facial esthetics in orthodontic planning (13).

By integrating multiple data modalities and extracting clinically significant patterns, AI enhances diagnostic accuracy and facilitates earlier interventions. This multimodal approach enables orthodontists to track treatment changes dynamically rather than relying on periodic static assessments, allowing for real-time adjustments to optimize patient outcomes (13).

### 2.2 Deep learning and AI-driven decision support

Deep learning has proven to be particularly powerful in predicting treatment outcomes, automating diagnosis, and optimizing clinical decision-making. Unlike traditional machine learning models that rely on manually defined features, deep learning algorithms autonomously learn complex anatomical structures and treatment patterns from vast datasets, improving their predictive accuracy over time (14).

A wide range of deep learning models have been developed and validated for various clinical tasks in orthodontics, ranging from diagnostic landmark detection to biomechanical force simulation and treatment planning. Table 1 provides a summary of representative AI models, highlighting their architectures, clinical applications, and performance metrics. These examples illustrate the breadth of deep learning applications and underscore the accelerating adoption of AI in orthodontic workflows.

**Table 1:** Representative AI models in orthodontic practice and evaluation.

Study	Model category	Task	Clinical application	Performance metrics
Wu et al. (19)	Two-stage Mesh DL (iMeshSegNet + PointNet-Reg)	3D Tooth Segmentation + Landmark Localization	Orthodontic treatment planning using intraoral scans	DSC = 0.964 ± 0.054; Landmark MAE = 0.597 ± 0.761 mm
Vinayahalingam et al. (20)	PointCNN + 3D U-Net	3D Tooth Segmentation + FDI Labeling	Used in Orthodontic planning	Segm. IoU = 0.915, Labeling Acc. = 0.894
Jiang et al. (21)	CephNet (2-stage CNN: RegionNet + LocationNet)	Cephalometric landmark localization	Auto cephalometric analysis across clinics and devices	Accuracy for cephalometric measurements SCR = 89.33%
Zhang et al. (15)	ResNet50-based CNN	Mandibular growth trend prediction (binary)	Predicting mandibular overgrowth in Class III	Acc. = 85%; Sens. = 0.95; Spec. = 0.75; AUC = 0.9775
Chen et al. (10)	Deep Learning (Multimodal fusion)	Multimodal feature fusion and classification for facial monitoring	Orthodontic monitoring combining 2D facial and 3D scan data	Improved feature extraction and classification
Li et al. (8)	PointNet-based Neural Network	Tooth-bracket segmentation + Feature extraction	Virtual bracket removal and bracket position assessment	Accuracy = 98.93% (Dataset A); 97.42% (Dataset B); Time = 2.9 ms/tooth
Xu et al. (22)	EfficientNet-B1–B5, MobileNet V3	OIERR (root resorption) grading on CBCT slices	Automated assessment of root resorption severity	AUC = 0.956; Accuracy = 95%; Specificity = 98%

One of the most impactful applications of deep learning in orthodontics is predicting mandibular growth patterns, which is critical for determining treatment strategies in pediatric patients. A deep learning-based CNN model trained on cephalometric images achieved 85% accuracy in predicting mandibular growth trends in children with anterior crossbite, significantly outperforming junior orthodontists (54.2%). The model identified key anatomical predictors, including chin morphology, condylar growth, and airway dimensions, providing early-stage intervention recommendations (15).

Beyond skeletal growth prediction, AI has also been integrated into digital smile simulation systems, allowing patients to visualize post-treatment outcomes before initiating therapy. AI-driven smile simulations assess facial proportions, lip support, and dental arch relationships, aiding in treatment planning (16). A study on Invisalign SmileView found that AI accurately predicted philtrum height, commissure width, and buccal corridor display but underestimated maxillary intercanine width and smile arc curvature, underscoring both the strengths and current limitations of AI in esthetic prediction (12).

Furthermore, AI plays a crucial role in biomechanical modeling for orthodontic force application. AI-driven finite element models (FEMs) simulate the biomechanical effects of orthodontic forces on teeth and surrounding structures, allowing for the optimization of aligner and bracket designs to enhance treatment efficacy (17).

By eliminating much of the subjectivity inherent in orthodontic decision-making, deep learning refines treatment planning and enhances predictive precision. As AI models continue to evolve, their applications in orthodontics will enable increasingly data-driven, patient-specific interventions (18).

### 2.3 Adaptive AI: real-time learning for orthodontic optimization

As shown in these examples, AI models are increasingly integrated into clinical decision-making. The next step in this evolution involves adaptive AI systems that respond dynamically to patient data in real time, as discussed in the following section.

The integration of AI in orthodontics extends beyond static treatment planning into adaptive learning, where AI continuously refines treatment strategies based on real-time patient data. Unlike traditional orthodontic workflows that rely on predefined milestones and periodic assessments, AI-driven systems analyze sequential imaging, intraoral scans, and biomechanical responses to detect subtle deviations from expected outcomes. These deviations may arise due to biological variability, mechanical inefficiencies, or patient compliance issues. By leveraging deep reinforcement learning, AI can self-optimize by recognizing these deviations and dynamically adjusting treatment plans to maintain efficiency and precision (23).

One critical area where adaptive AI plays a transformative role is in orthodontic force optimization. Orthodontic tooth movement depends on the precise application of biomechanical forces, and excessive or improperly directed forces can lead to complications such as root resorption, periodontal damage, or inefficient tooth movement. AI-powered FEMs simulate the biomechanical effects of orthodontic forces, allowing for real-time optimization of bracket positioning, aligner sequencing, and force application (24). By analyzing patterns in treatment progress, AI can predict root movement trajectories, identify regions of excessive stress, and recommend strategic adjustments that enhance treatment efficiency while minimizing adverse effects (17).

Predictive modeling further enhances the adaptability of AI-driven orthodontic systems. By continuously analyzing a patient's response to orthodontic forces, AI can anticipate challenges before they arise. This predictive capability allows orthodontists to make preemptive adjustments, such as modifying force vectors, repositioning anchorage points, or adjusting appliance configurations to prevent complications. The ability to model and simulate treatment outcomes dynamically helps optimize decision-making and reduces reliance on reactive interventions, ultimately improving treatment precision and predictability.

The teledentistry platform integrates AI prediction models such as Long Short-Term Memory (LSTM) networks through API interfaces to support multidisciplinary teams to dynamically adjust treatment plans during virtual consultations. For example, when the AI predicts that the patient may derail the orthodontic appliance due to a growth surge, the system automatically triggers a consultation request, the general dentist uploads high-precision intraoral scanning data (with a resolution of ±20 μm), and the orthodontic specialist evaluates the jawbone growth trend and mechanical distribution through 3D visualization tools to make collaborative decisions on follow-up appointment times or orthodontic force adjustments (15).

Adaptive AI represents a paradigm shift in orthodontics, transitioning from static, pre-planned treatment strategies to dynamically evolving, real-time adjustments. By leveraging continuous feedback loops, force optimization models, predictive analytics, and remote monitoring integration, AI enhances treatment efficiency, reduces complications, and personalizes patient care (25). As AI continues to evolve, its ability to self-learn from patient-specific responses will further revolutionize orthodontic treatment, setting new standards for precision and adaptability.

## 3 AI-driven orthodontic treatment planning and prediction

AI-driven remote monitoring improves aligner adherence by predicting the risk of derailment. Wafaie et al. (3) analyzed sensor data from 1,200 patients and used machine learning to predict aligner displacement with 82% accuracy, resulting in a 28% reduction in unscheduled follow-up visits. In addition, Hung et al. (26) found that women and younger patients were more likely to initiate treatment, but no demographic factors significantly affected long-term adherence.

Unlike conventional planning, which relies on periodic adjustments, AI continuously refines treatment strategies based on real-time patient progress. This not only improves treatment predictability but also minimizes complications, reduces treatment duration, and enhances patient satisfaction. Additionally, AI facilitates interdisciplinary collaboration, assisting in complex cases that require surgical or prosthetic interventions. As AI technology evolves, its predictive capabilities will further streamline orthodontic planning, making treatment more efficient and outcome-focused (23).

### 3.1 Predictive modeling for treatment optimization

AI has revolutionized orthodontic treatment planning by introducing predictive modeling techniques that improve diagnostic precision and clinical decision-making before treatment begins. Traditional orthodontic planning relies on cephalometric analysis, clinical experience, and static imaging to evaluate skeletal and dental relationships. However, AI-driven predictive models integrate vast datasets, automate cephalometric landmark detection, and simulate skeletal growth trends, allowing for more precise and individualized orthodontic interventions before any appliance is placed (27).

One of the most significant applications of AI in orthodontics is growth prediction modeling, particularly in pediatric and adolescent patients. Deep learning-based algorithms trained on cephalometric images can analyze mandibular growth patterns, craniofacial development, and occlusal changes over time, aiding clinicians in deciding whether orthopedic correction, fixed appliances, or even orthognathic surgery may be necessary (28). AI also plays a critical role in tooth movement prediction, forecasting how different orthodontic forces will influence individual teeth and the surrounding bone structures. Additionally, AI-driven cephalometric landmark detection systems provide automated skeletal and dental analyses with high accuracy, reducing variability among clinicians and expediting treatment planning. Recent advancements in AI-powered occlusal classification have enabled more precise determination of malocclusion severity, helping orthodontists tailor treatment strategies based on patient-specific biomechanical responses (6).

By leveraging these predictive capabilities, orthodontists can customize treatment plans before active intervention begins, reducing the risk of inefficiencies and mid-treatment complications. While predictive modeling sets the foundation for treatment, real-time virtual scenario testing ensures that orthodontic plans evolve dynamically based on actual patient response, as discussed in the next section (29).

### 3.2 Virtual scenario testing and digital treatment simulation

AI-based virtual scenario testing provides continuous feedback during orthodontic treatment, enabling orthodontists to adjust treatment dynamically based on real-time patient response. Unlike predictive modeling, which sets pre-treatment expectations, real-time AI simulations refine treatment mid-course, ensuring aligner sequencing, bracket positioning, and force application remain optimal throughout the process (30).

This technology leverages digital twin modeling, where a virtual representation of the patient's dentition undergoes simulated orthodontic forces before real-world application. AI can compare actual vs. predicted tooth movement using intraoral scans, CBCT imaging, and occlusal force distribution models, allowing orthodontists to modify appliance adjustments dynamically. For example, if AI detects delayed tooth movement in aligner therapy, it can recommend refinements to force vectors or treatment timelines to prevent treatment stagnation (31).

In bracket-based treatment, AI-driven FEMs assist in optimizing biomechanical force distribution. These simulations predict how teeth will respond to different torque, angulation, and extrusion forces, helping orthodontists refine bracket placements to minimize unwanted movements such as tipping or root resorption (32).

Additionally, real-time AI simulations help manage mid-treatment complications, such as detecting early signs of posterior bite collapse, excessive incisor proclination, or insufficient occlusal contact. AI-assisted monitoring tools, including smart intraoral scanning systems, allow orthodontists to track treatment progress remotely, ensuring more precise and patient-specific refinements.

By incorporating AI-driven iterative scenario testing, orthodontic treatment becomes more adaptive, reducing reliance on manual adjustments while improving treatment precision and predictability. This continuous feedback loop between AI simulations and real-world patient response represents a significant advancement in modern orthodontic care.

### 3.3 AI in risk management: early detection and intervention

Orthodontic treatment carries inherent risks, including external root resorption (ERR), periodontal disease, and alveolar bone loss, which can significantly impact treatment outcomes. Traditional risk assessment methods rely on periodic radiographic examinations and clinical evaluations, which may delay the detection of early-stage complications. AI-powered risk assessment models, particularly those based on machine learning (ML) and DL, offer an innovative approach by identifying risks earlier, predicting potential complications, and optimizing treatment strategies (33).

ERR is a major concern in orthodontics, particularly in patients undergoing fixed appliance treatment. Studies show that ERR affects up to 66% of orthodontic patients, with severe cases leading to irreversible root shortening and compromised tooth stability (34). AI-based CBCT analysis has demonstrated superior accuracy in detecting early-stage ERR compared to conventional radiographic assessment (35). Deep learning models, particularly CNNs, have shown over 90% accuracy in detecting minor resorptive changes that are often missed by human evaluators (36). Furthermore, hybrid AI models combining feature selection techniques (FST) with deep learning algorithms have enhanced diagnostic precision, achieving an AUC of 96% in ERR classification (37). This enables orthodontists to identify at-risk teeth before significant root damage occurs, allowing for timely treatment adjustments such as force modulation or treatment pauses (38).

Periodontal complications, including gingival inflammation, alveolar bone loss, and periodontal pocket formation, are significant risks during orthodontic treatment. The presence of fixed appliances increases biofilm accumulation, leading to higher susceptibility to periodontal diseases (39). AI-powered diagnostic systems have revolutionized periodontal health monitoring by using computer vision models to analyze intraoral images and CBCT scans for early signs of inflammation and bone loss. Deep learning algorithms, such as GoogLeNet and ResNet, have achieved over 94% accuracy in detecting gingivitis from intraoral images, outperforming manual clinical assessments (40). Additionally, AI-based cross-temporal multimodal imaging fusion techniques allow for radiation-free tracking of periodontal health, reducing reliance on frequent CBCT scans while maintaining diagnostic precision (10).

AI-driven risk management in orthodontics is revolutionizing early detection, predictive modeling, and real-time monitoring. By leveraging deep learning for ERR detection, periodontal health tracking, and treatment stability forecasting, AI allows orthodontists to shift from a reactive approach to a proactive, preventive strategy. This enhances treatment safety, efficiency, and long-term patient outcomes, making orthodontic care more precise and data-driven. Future advancements will continue refining AI applications, ensuring safer, more personalized orthodontic treatments (41).

## 4 Enhancing patient experience with AI

AI is not only transforming the clinical aspects of orthodontic treatment but also significantly enhancing the patient experience. By integrating AI-powered tools into patient management, orthodontists can provide a more engaging, transparent, and personalized treatment journey. AI-driven platforms improve communication, boost compliance, and allow for seamless remote monitoring, ensuring that patients remain actively involved in their treatment while reducing unnecessary in-office visits.

### 4.1 AI-Enabled patient engagement and compliance

Maintaining patient compliance with oral hygiene protocols and treatment recommendations remains a critical challenge. A randomized controlled trial (RCT) by Santonocito et al. demonstrated that AI chatbots reduced gingival inflammation (MGI: 15% improvement vs. control, *P* < 0.05) but showed no significant difference in treatment knowledge acquisition (42). Additionally, Kalaoglu et al. found that Invisalign First aligners had higher patient acceptance compared to traditional removable acrylic appliances (*P* = 0.014), highlighting the role of appliance design in compliance (43).

AI-driven orthodontic platforms allow patients to track their treatment progress in real time, offering visual representations of tooth movement, estimated completion timelines, and upcoming milestones. These tools enhance patient motivation and understanding by providing clear, tangible insights into their orthodontic journey. By seeing projected outcomes and real-time adjustments, patients gain confidence in their treatment and are more likely to adhere to prescribed protocols (44).

AI-based systems also play a crucial role in improving patient compliance by automating reminders and providing personalized guidance. For instance, AI-powered apps can send alerts for aligner wear time, remind patients about upcoming appointments, and offer step-by-step oral hygiene recommendations. Furthermore, AI-driven chatbots and virtual assistants can answer common patient questions, providing 24/7 support without requiring direct intervention from orthodontists. By integrating AI into patient education, orthodontic practices can ensure that patients remain well-informed and engaged throughout their treatment (32).

### 4.2 Resource identification initiative

The integration of AI into orthodontic care has paved the way for remote treatment tracking and virtual consultations, significantly reducing the need for frequent in-office visits. This approach is particularly beneficial for long-term orthodontic treatments, where continuous monitoring and timely interventions are essential for maintaining progress and achieving optimal results (45).

AI-powered mobile applications and smart intraoral scanning devices enable patients to capture and upload images of their teeth at regular intervals. These AI-driven systems analyze the images and compare them with predicted treatment models, identifying any deviations or issues in real time (46). If any discrepancies arise, orthodontists receive instant notifications, allowing them to adjust treatment plans proactively. This remote monitoring capability not only increases efficiency but also minimizes the inconvenience of unnecessary clinic visits while ensuring that treatment stays on track.

AI-driven virtual consultations enhance patient accessibility to orthodontic care by offering real-time guidance and monitoring. Virtual assistants powered by AI can provide treatment-related advice, appointment reminders, and self-monitoring tips, ensuring patients remain actively engaged between visits. Additionally, AI-facilitated teleorthodontic consultations allow orthodontists to assess treatment progress remotely, reducing wait times and improving overall patient convenience.

By integrating AI into patient management and treatment monitoring, orthodontic care becomes more accessible, efficient, and patient-centered. AI-powered platforms not only enhance engagement and compliance but also improve treatment accuracy through continuous, real-time monitoring (47). As AI technology continues to evolve, its role in patient experience will further expand, bridging the gap between in-office visits and digital healthcare solutions.

## 5 Challenges and future directions in AI-driven orthodontics

As AI continues to revolutionize orthodontic treatment, its widespread adoption faces several technical and clinical challenges. While AI offers immense potential in improving treatment precision, patient experience, and efficiency, it also raises concerns regarding data security, algorithm reliability, and the need for clinical validation. Additionally, future advancements in AI-driven orthodontics will depend on interdisciplinary integration and innovations in smart orthodontic appliances.

### 5.1 Technical and ethical considerations

Despite AI's growing presence in orthodontics, certain technical limitations must be addressed to ensure its seamless integration into clinical practice (23).

AI systems rely on large volumes of patient data, including radiographs, intraoral scans, and electronic health records, to train and refine their predictive models. However, the use of such sensitive information raises concerns about data privacy and cybersecurity. Ensuring secure encryption, adherence to regulations such as HIPAA and GDPR, and ethical handling of patient data is essential to maintain trust and compliance in AI-driven orthodontic solutions. Future AI models must prioritize data protection through advanced anonymization techniques and decentralized processing to minimize security risks (48).

A major challenge in AI-based orthodontic treatment planning is the lack of transparency in decision-making, often referred to as the “black box” effect. Many deep learning algorithms generate treatment recommendations without clearly explaining the rationale behind them, making it difficult for clinicians to fully trust and interpret AI-driven insights. To enhance reliability, researchers must develop explainable AI (XAI) models that provide interpretable reasoning for each prediction, allowing orthodontists to verify and validate AI-generated recommendations. Increasing algorithm transparency will not only improve clinician trust but also facilitate regulatory approvals and ethical adoption of AI in orthodontics (49).

### 5.2 Clinical validation and integration into practice

For AI to become a standard tool in orthodontics, it must be rigorously tested and validated through clinical trials and real-world applications (50).

AI should be positioned as an assistant rather than a replacement for orthodontists. The role of AI is to provide real-time, data-driven insights that enhance clinical decision-making, while human expertise remains central to treatment planning and patient care (51). The integration of AI into orthodontics should focus on improving workflow efficiency, reducing diagnostic errors, and supporting personalized treatment rather than replacing clinical judgment. Striking a balance between AI automation and human oversight is crucial to ensuring optimal patient outcomes (21).

Before AI-powered orthodontic tools can be widely adopted, they must undergo rigorous clinical validation to prove their safety, accuracy, and efficacy. Large-scale clinical trials and real-world studies are needed to assess AI's performance across diverse patient populations, treatment modalities, and clinical conditions (19). Furthermore, AI models must be continuously refined based on real-world feedback to enhance their adaptability to individual patient variations. Regulatory agencies and professional orthodontic organizations must establish standardized guidelines for evaluating AI-driven orthodontic systems, ensuring that they meet the highest clinical and ethical standards (20).

### 5.3 Future research and smart orthodontic technologies

The future of AI in orthodontics lies in expanding its capabilities through interdisciplinary collaboration and the development of intelligent orthodontic devices (52).

To maximize AI's potential, future research should explore its integration with other fields, such as genomics, biomechanics, and dental materials science (53). By incorporating genetic factors into orthodontic treatment planning, AI could predict individual responses to orthodontic forces more accurately. Similarly, combining AI with biomechanical simulations could lead to better force distribution models, optimizing treatment efficiency while minimizing risks such as root resorption. Additionally, advancements in dental materials enhanced by AI-driven design could lead to the development of more effective and durable orthodontic appliances (35).

AI has the potential to revolutionize orthodontic hardware by introducing smart appliances that adapt to patient-specific needs. Hung M et al.'s (26) cross-sectional study showed that being female (OR = 1.38, 95% CI = 1.12–1.70, *P* = 0.002) and being <25 years old (OR = 1.54, 95% CI = 1.22–1.95, *p* < 0.001) were significant predictors of willingness to initiate orthodontic treatment; however, the study did not analyze the association between demographic characteristics and long-term orthodontic appliance compliance. Lei et al. (54) systematically reviewed 48 AI facial analysis studies (2018–2024) and noted that CNNs were 89% (95% CI = 86%–92%) accurate in predicting trends in soft tissue changes after orthognathic surgery. Kengne Talla et al. (55) analyzed 32 teledentistry studies and showed that teleconsultations increased caries screening participation by 34% (*P* = 0.013), but only five studies involved orthodontic telemonitoring, and all had small sample sizes. However, demographic correlates of long-term adherence, validation of orthodontic telemonitoring at scale still need to be explored in depth, and future studies need to incorporate intelligent tools and humanistic strategies for personalized treatment management.

While AI has already demonstrated its transformative potential in orthodontics, addressing technical challenges and ensuring robust clinical validation are crucial for its long-term success. Enhancing algorithm transparency, strengthening data security, and refining AI-human collaboration will pave the way for more widespread adoption (53). Future interdisciplinary research and innovations in smart orthodontic appliances will further push the boundaries of AI-driven treatment, bringing orthodontics into a new era of precision, efficiency, and patient-centered care. With continued advancements, AI is poised to become an indispensable tool in modern orthodontic practice, shaping the future of personalized and data-driven treatment solutions.

## 6 Conclusion

AI has redefined orthodontic treatment by shifting from static, periodic evaluations to a dynamic, real-time management system that enhances precision, efficiency, and patient engagement. By integrating advanced imaging analysis, predictive modeling, and remote monitoring tools, AI empowers orthodontists to make data-driven decisions, personalize treatment plans, and mitigate risks proactively. While challenges remain in ensuring algorithm transparency, clinical validation, and data security, ongoing research and technological advancements will drive the integration of AI into mainstream orthodontic practice. As AI continues to evolve, its role in orthodontics will expand, paving the way for more intelligent, adaptive, and patient-centered treatment solutions.
